# SRSF1/Mcl‐1 Axis Drives Apoptosis Evasion and Shapes the Immune Microenvironment to Promote Gastric Cancer Progression

**DOI:** 10.1155/humu/9554600

**Published:** 2026-06-10

**Authors:** Xingguang Liu, Deming Liu, Guangming Zhang, Shuo Liu, Hui Cai

**Affiliations:** ^1^ The First School of Clinical Medicine, Lanzhou University, Lanzhou, China, lzu.edu.cn; ^2^ NHC Key Laboratory of Diagnosis and Therapy of Gastrointestinal Tumor, Gansu Provincial Hospital, Lanzhou, China, gsyy.cn; ^3^ Gansu Key Laboratory of Molecular Diagnostics and Precision Medicine for Surgical Oncology, Lanzhou, China; ^4^ Key Laboratory of Molecular Diagnostics and Precision Medicine for Surgical Oncology in Gansu Province, Gansu Provincial Hospital, Lanzhou, China, gsyy.cn; ^5^ Department of Great Vessel Surgery, Gansu Provincial Hospital, Lanzhou, China, gsyy.cn

**Keywords:** apoptosis, gastric cancer (GC), Mcl-1, metastasis, SRSF1, tumor microenvironment (TME)

## Abstract

Gastric cancer (GC) is one of the malignancies with the highest incidence and mortality worldwide. Evasion of apoptosis is a hallmark of cancer that drives tumor progression. The splicing factor SRSF1 and antiapoptotic protein Mcl‐1, including its isoforms Mcl‐1L and Mcl‐1S, play significant roles in cancer development; however, the regulatory mechanisms of the SRSF1‐Mcl‐1 axis in GC remain unclear. This study systematically evaluated the function of SRSF1 in GC by integrating multidatabase analyses (TIMER, UALCAN, and KM‐Plotter), in vitro experiments (qRT‐PCR, Western blot, Transwell migration/invasion, and apoptosis assays), in vivo xenograft models, and bioinformatic approaches (single‐cell RNA sequencing, hdWGCNA, cell interaction analysis, and mutational analysis). SRSF1 is significantly overexpressed in GC tissues and cell lines, correlating with poor prognosis in patients. Through comprehensive multiomics analysis, we first revealed that SRSF1‐positive malignant epithelial cells possess a unique coexpression network and exhibit significantly enhanced interactions with fibroblasts, reshaping the tumor microenvironment (TME). Subsequently, functional assays demonstrated that SRSF1 overexpression enhances cell invasion, migration, and apoptosis resistance by inhibiting the proapoptotic isoform Mcl‐1S and suppressing the mitochondrial apoptosis pathway (Bak/caspase‐9/caspase‐3). In conclusion, the SRSF1‐Mcl‐1 axis serves as a dual key regulator of invasion/migration and apoptosis evasion in GC, providing a new strategy for targeted therapy in advanced GC.

## 1. Introduction

Gastric cancer (GC) imposes a significant global public health burden. It ranks among the leading cancers worldwide in both incidence and mortality [1]. In countries like Japan and South Korea, widespread screening programs have facilitated early detection and improved prognosis. In contrast, GC is frequently diagnosed at an advanced stage in many countries. This pattern consistently drives poor long‐term survival [2]. There is a critical need to elucidate GC pathogenesis and develop more effective early diagnostic and therapeutic strategies.

Cancer progression entails tumor invasion and metastasis, driven by cumulative genetic alterations in regulators of cell proliferation, mitosis, invasiveness, angiogenesis, and apoptosis suppression [3]. Critically, accumulating evidence underscores the central role of apoptotic evasion in driving tumor invasion and metastatic dissemination [4]. The Bcl‐2 protein family comprises key regulators of the mitochondrial apoptosis pathway. Within this family, myeloid cell leukemia‐1 (Mcl‐1) functions as a potent antiapoptotic protein: Its overexpression is observed across diverse human cancers and strongly correlates with tumor progression, therapeutic resistance, and poor clinical outcomes [5, 6]. Previous studies have shown that Mcl‐1 is overexpressed in GC tissues and significantly correlates with advanced tumor stage, metastasis, and poor patient survival [7, 8]. Mcl‐1 sustains cancer cell survival by blocking the activation of key proapoptotic proteins in the mitochondrial apoptosis pathway (e.g., Bax/Bak) and acts as a critical mediator of chemoresistance [6]. Our previous studies have further demonstrated that distinct alternative splicing patterns of Mcl‐1 exist in GC tissues and are closely associated with both aggressive clinical course and poor patient prognosis [9].

Mcl‐1 encodes two functional isoforms: antiapoptotic Mcl‐1L and proapoptotic myeloid cell leukemia‐1 short isoform (Mcl‐1S). An imbalance in their ratio is a core mechanism underlying apoptosis evasion [6]. Recent studies have shown that Mcl‐1 isoform expression is tightly regulated by splicing factors [10, 11]. Among these, serine/arginine‐rich splicing factor 1 (SRSF1)—a key RNA‐binding protein—recognizes specific cis‐elements in pre‐mRNA to dictate exon skipping or inclusion, thereby controlling isoform switching in target genes [11]. Empirical evidence indicates that SRSF1 overexpression promotes the production of tumor‐promoting isoforms, thereby enhancing proliferation, metastasis, and apoptosis resistance in multiple cancers [10, 11]. However, whether SRSF1 regulates Mcl‐1 isoform switching to drive apoptosis evasion in GC remains inconclusive.

This study first elucidates the regulatory mechanism of the SRSF1‐Mcl‐1 axis in GC by uncovering the molecular basis of cellular evasion of apoptosis and identifying new therapeutic targets. Secondly, through bioinformatic approaches, including single‐cell RNA sequencing, high‐dimensional weighted gene coexpression network analysis (hdWGCNA), cell interaction analysis, and mutational analysis, the study systematically assesses the function of SRSF1 in GC and its impact on the tumor microenvironment (TME). These findings lay the groundwork for strategies to improve prognosis, particularly for patients with advanced GC.

## 2. Methods

### 2.1. Databases for Differential Expression and Survival Analysis

The expression profile of SRSF1 was analyzed across 32 tumor types in the TCGA database using the TIMER platform, assessing its correlation with immune cell infiltration [12]. UALCAN was utilized to examine the relationship between SRSF1 transcription levels and clinical characteristics of gastric adenocarcinoma patients (TCGA‐STAD cohort) [13]. Prognostic validation was performed using the Kaplan–Meier plotter, evaluating overall survival (OS) with median expression thresholds and multivariable Cox regression [14].

### 2.2. Cell Acquisition and Culture Conditions

Human gastric epithelial cells GES‐1 and GC cell lines (AGS, HGC27, MKN28, MKN45, and SNU‐1) were cultured in RPMI‐1640 or MEM medium containing 10% fetal bovine serum, penicillin (0.1 mg/mL), and streptomycin (0.1 mg/mL) at 37°C in a 5% CO_2_ incubator. Cell cryopreservation was performed according to supplier protocols.

### 2.3. Lentiviral Transduction and Validation

SRSF1 overexpression was achieved using the pSLenti‐EF1‐EGFP‐Puro vector. Knockdown was conducted using three shRNAs targeting SRSF1 CDS (sequences listed in Table S1) and cloned into the pSLenti‐U6‐shRNA‐CMV‐Puro vector. Cells were transduced at 70% confluency and selected with puromycin (2 *μ*g/mL). Validation was performed via quantitative real‐time PCR (qRT‐PCR), Western blot, and fluorescence microscopy.

### 2.4. qRT‐PCR

Total RNA was extracted using the TRIzol method, followed by DNase I digestion to remove genomic DNA and cDNA synthesis using the Hifair III kit. qRT‐PCR was performed using SYBR Green Mix with the following cycling conditions: 95°C for 5 min; 40 cycles (95°C for 10 s, 60°C for 20 s, and 72°C for 20 s); and a final extension at 95°C for 10 s. Primers were synthesized by Sangon Biotech (sequences in Table S2), and relative expression was calculated using the 2^−*ΔΔ*Ct^ method, normalized to GAPDH.

### 2.5. Western Blotting

Proteins were extracted from cells using RIPA buffer and quantified using the BCA method. After denaturation at 95°C, 30 *μ*g of protein was separated via 10% SDS‐PAGE and transferred to PVDF membranes. Membranes were blocked in 5% nonfat milk for 1 h and incubated with primary antibodies (SRSF1, Mcl‐1, BAK1, cleaved caspase‐9, cleaved caspase‐3, and GAPDH) at 4°C, followed by HRP‐conjugated secondary antibodies at room temperature for 1 h. Bands were visualized with ECL, quantified using ImageJ, and normalized to GAPDH.

### 2.6. Colony Formation and CCK‐8 Assay

Cells were seeded at 500–1000 cells/well in 6‐well plates and cultured for 14 days. Colonies were fixed with 4% paraformaldehyde, stained with crystal violet, and counted if they had more than 10 cells. Cell viability was assessed using the CCK‐8 assay. Cells were incubated with CCK‐8 reagent after 24/48/72 h, and absorbance at 450 nm was measured.

### 2.7. Transwell Migration and Invasion Assay

Migration assays utilized Transwell chambers with 8 *μ*m polycarbonate membranes; invasion assays were precoated with 2.5% Matrigel. Cells were serum‐starved for 12 h and seeded into the upper chamber at 1 × 10^5^ cells/mL, with 10% FBS medium in the lower chamber. Migration was incubated for 24 h and invasion for 48 h. Cells in the upper chamber were removed with cotton swabs, while cells in the lower chamber were fixed with 4% paraformaldehyde, stained with crystal violet, and counted using ImageJ across five fields.

### 2.8. Flow Cytometry for Apoptosis and Cell Cycle

Cells were stained with Annexin V‐APC/7‐AAD apoptosis detection kit and analyzed for apoptosis rates using flow cytometry. Cell cycle analysis was performed using a DNA staining kit, PI staining, and flow cytometry, and ModFit software was used to assess the G_0_/G_1_, S, and G_2_/M phases.

### 2.9. Xenograft Model Establishment

BALB/c nude mice (4–6 weeks old) were subcutaneously injected with stably transduced cells (5 × 10^6^ cells/mouse), with weight and tumor volume (*V* = length × width^2^/2) monitored biweekly. After 4 weeks, mice were sacrificed, tumors were weighed, and tissues were cryopreserved.

### 2.10. Immunohistochemical (IHC) Analysis

Paraffin sections were subjected to antigen retrieval, endogenous peroxidase blocking, and incubation with primary antibodies (Bak, cleaved caspase‐3, and cleaved caspase‐9) at 4°C, followed by incubation with secondary antibodies at room temperature for 30 min. DAB was used for staining, with hematoxylin counterstaining. ImageJ was employed to quantify integrated optical density across five random fields.

### 2.11. Statistical Analysis

Data were analyzed using SPSS 22.0 and GraphPad Prism 8.5. All in vitro experiments (including qRT‐PCR, Western blot, colony formation, CCK‐8, Transwell, and flow cytometry) were performed in triplicate (*n* = 3 independent experiments). Normally distributed data were evaluated with one‐way ANOVA and LSD‐t tests; nonnormally distributed data were analyzed with non‐parametric tests. Significance was set at *p* < 0.05.

### 2.12. scRNA‐seq Analysis

UMI matrices from nine gastric biopsy samples (five normal and four tumor) were downloaded from the GEO dataset GSE167297. Data were rescaled using Seurat (v4.3.1), regressed for mitochondrial content, and the Top 2000 variable genes were identified. PCA was followed by clustering with the Top 30 components and UMAP dimensional reduction, defining cell clusters based on marker genes (Wilcoxon test). Malignant epithelial cells were distinguished from nonmalignant epithelial populations based on the expression of known epithelial malignancy markers (e.g., EPCAM and KRT8) and confirmed by estimating large‐scale chromosomal copy number variations (CNVs) using the inferCNV package.

### 2.13. hdWGCNA

Malignant epithelial cells were categorized into SRSF1‐positive and negative groups based on SRSF1 UMI levels, and a coexpression network was constructed using the hdWGCNA package. The soft‐threshold was selected to satisfy the scale‐free topology, the topological overlap matrix (TOM) was computed, and modules were identified using dynamic tree cut (minimum 30 genes). Module eigengenes were correlated with SRSF1 status, followed by GO enrichment analysis.

### 2.14. Cell–Cell Interaction Analysis

The CellChat package (v1.6.0) was used to compare signaling interactions among SRSF1‐positive and negative cells and other groups in tumor and normal tissues. CellChat objects were created separately to infer ligand–receptor pairs visualizing differential pathways and interaction strengths.

### 2.15. Mutational Analysis

MAF files from the TCGA‐STAD cohort were obtained from GDC and processed using the maftools package (v2.18.0). Mutational burden, spectrum, and variant classification were analyzed, with hotspot mutations visualized using lollipopPlot2.

## 3. Results

### 3.1. Multidatabase and Experimental Validation Reveal High Expression of SRSF1 Correlates With Poor Prognosis in GC

**Figure 1:**
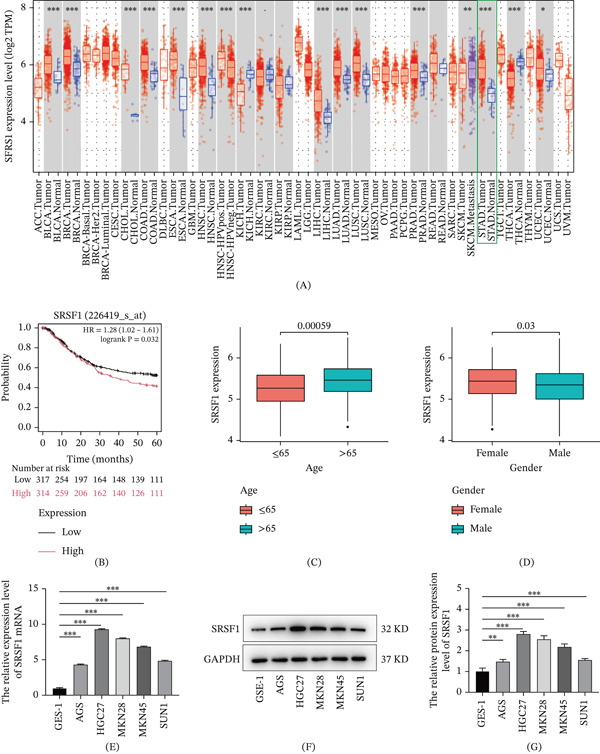
SRSF1 is upregulated in gastric cancer and associated with poor prognosis. (A) UALCAN database analysis showing SRSF1 expression levels in multiple solid tumors (gastric adenocarcinoma, bladder urothelial carcinoma, breast cancer, and cholangiocarcinoma) compared to matched normal tissues. (B) Kaplan–Meier plotter analysis of overall survival (OS) in gastric cancer (GC) patients stratified by SRSF1 expression level (high vs. low), with hazard ratio (HR = 1.28, *p* = 0.032). (C) Comparison of SRSF1 expression in GC patients aged > 65 years versus younger cohorts (*p* = 0.00059). (D) SRSF1 expression in female versus male GC patients (*p* = 0.03). (E) qRT‐PCR analysis of SRSF1 mRNA levels in five GC cell lines (AGS, HGC27, MKN28, MKN45, and SNU1) compared to normal gastric mucosal cells (GSE). (F, G) Western blot analysis of SRSF1 protein levels in the same GC cell lines versus GSE control. ∗ indicates *p* < 0.05; ∗∗ indicates *p* < 0.01; ∗∗∗ indicates *p* < 0.001.

Integrated analysis of the TIMER, UALCAN, and KM plotter databases revealed that SRSF1 expression was significantly upregulated in multiple solid tumors, including gastric adenocarcinoma, bladder urothelial carcinoma, breast cancer, and cholangiocarcinoma, compared to their matched normal tissues (*p* < 0.05, Figure 1A,B). Analysis of GC cohort data showed that patients aged > 65 years had ~37% higher SRSF1 expression than younger cohorts (*p* = 0.00059, Figure 1C). Expression was also significantly elevated in females versus males (*p* = 0.03, Figure 1D). Survival analysis revealed significantly lower 5‐year OS rates in patients with high SRSF1 expression (HR = 1.28, *p* = 0.032, Figure 1B), supporting SRSF1 as a potential prognostic biomarker. Finally, experiments on GC cell lines (AGS, HGC27, MKN28, MKN45, and SNU1) consistently demonstrated elevated SRSF1 mRNA/protein via qRT‐PCR and Western blot, compared to normal gastric mucosal lines (GSE) (Figure 1E–G).

### 3.2. Characterization of SRSF1‐Positive Malignant Epithelial Cells

**Figure 2:**
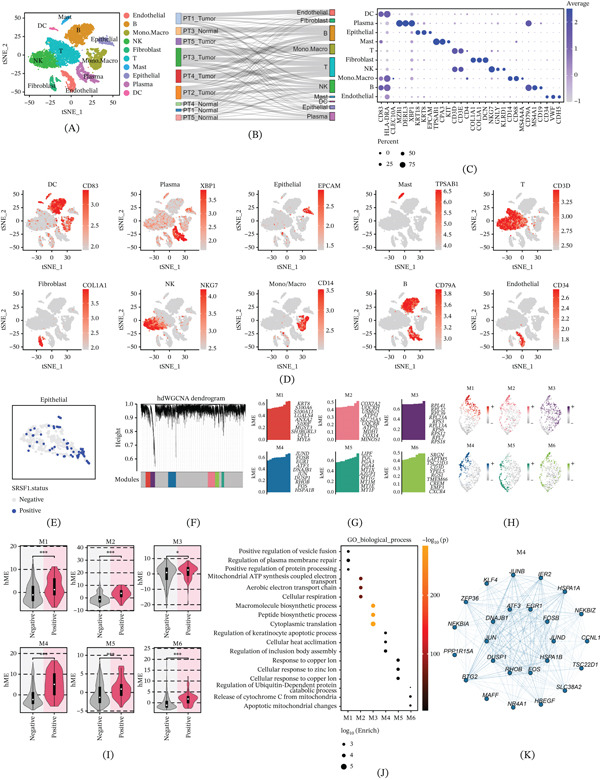
Characterization of SRSF1‐positive malignant epithelial cells in gastric cancer. (A) t‐SNE projection of 12,785 single cells from gastric cancer samples, revealing 10 major cell subpopulations: endothelial cells, B cells, monocytes/macrophages, NK cells, fibroblasts, T cells, mast cells, epithelial cells, plasma cells, and dendritic cells. (B) Sankey diagram illustrating the cellular distribution across individual samples. (C) Dot plot showing the expression of established marker genes used for cluster identification and validation. (D) Feature plots displaying the distribution of subtype‐specific markers across the cellular landscape. (E) t‐SNE projection of 545 malignant epithelial cells stratified by SRSF1 expression, distinguishing SRSF1‐positive (blue) and SRSF1‐negative (gray) subsets. (F) Identification of six distinct gene modules through high‐dimensional weighted gene coexpression network analysis (hdWGCNA) on malignant epithelial cells. (G) Module membership (kME) ranking of genes within each of the six identified modules. (H) Visualization of gene expression patterns across the six hdWGCNA modules. (I) Comparison of hub module eigengene (hME) scores between SRSF1‐positive and SRSF1‐negative malignant epithelial cells, with significant differences indicated ( ^∗^ *p* < 0.05,  ^∗∗^ *p* < 0.01, and  ^∗∗∗^ *p* < 0.001). (J) Gene Ontology enrichment analysis assigns unique biological functions to each module. (K) Network visualization of Module 4 (M4), the module most strongly correlated with the SRSF1‐positive phenotype, highlighting core gene coexpression interactions.

Single‐cell RNA sequencing analysis of 12,785 cells from GC samples delineated a comprehensive TME composed of 10 major cell subpopulations, including Endothelial cells, B cells, monocytes/macrophages, natural killer (NK) cells, fibroblasts, T cells, mast cells, epithelial cells, plasma cells, and dendritic cells, as visualized by t‐SNE projection (Figure 2A), with cellular distribution across samples further detailed by a Sankey diagram (Figure 2B); the identity of each cluster was validated by marker gene expression (Figure 2C), and subtype‐specific marker distribution was mapped across the cellular landscape (Figure 2D). From this atlas, 545 malignant epithelial cells were isolated and stratified by SRSF1 expression, revealing distinct SRSF1‐positive and SRSF1‐negative subsets within the t‐SNE space (Figure 2E). Subsequent hdWGCNA on these malignant cells identified six distinct gene modules (Figure 2F), with genes ranked by module membership (kME) within each (Figure 2G) and their expression patterns visually mapped (Figure 2H). Comparative analysis demonstrated significant differences in hub module eigengene (hME) scores for specific modules between SRSF1‐positive and SRSF1‐negative cells (Figure 2I), and Gene Ontology enrichment attributed unique biological functions to each module (Figure 2J). Notably, Module 4 (M4) emerged as the network most strongly correlated with the SRSF1‐positive phenotype, and its core gene coexpression architecture was visualized to highlight key functional interactions (Figure 2K).

### 3.3. Interaction Landscape Analysis Between SRSF1+ Malignant Epithelial Cells and Other Cellular Subpopulations in Normal and Cancer Tissues

**Figure 3:**
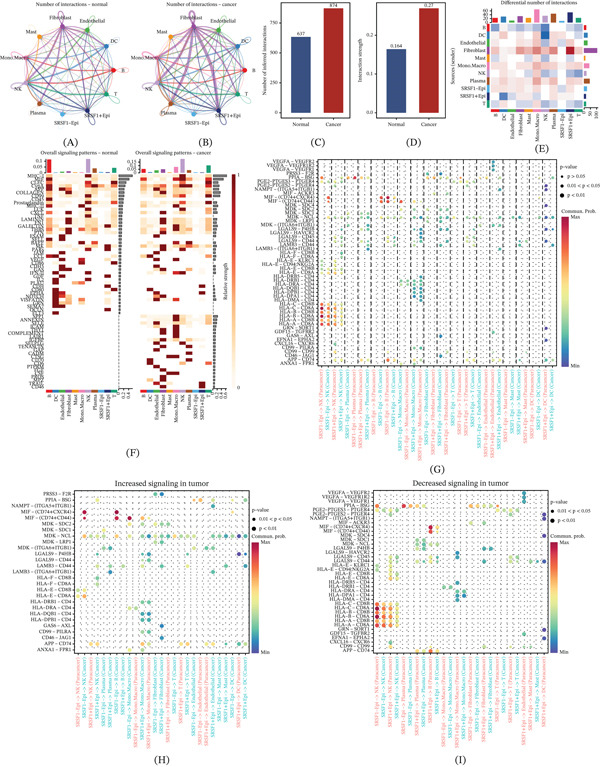
Interaction landscape between SRSF1‐positive malignant epithelial cells and other cellular subpopulations in normal and tumor tissues. (A, B) Cell–cell interaction networks in (A) normal and (B) tumor samples, with all cell populations labeled. (C, D) Bar charts quantifying the (C) number and (D) strength of cell–cell interactions, showing significantly elevated communication in tumors compared to normal tissues. (E) Heat map revealing frequent and strong interactions between SRSF1‐positive malignant epithelial cells and fibroblasts. (F) Differential signaling patterns between cell subpopulations in normal versus cancer settings. (G) Comparison of outgoing ligand‐receptor signal strength from SRSF1‐positive malignant epithelial cells to various receptor cell subpopulations in normal and tumor conditions. (H, I) Bubble plots detailing dysregulated communication in cancer, with (H) upregulated and (I) downregulated ligand‐receptor pairs.

Cell–cell interaction networks provide a comprehensive overview of cell–cell interactions in normal and tumor samples (Figure 3A,B), with all cell populations labeled. Bar charts show that the cell–cell interactions were significantly elevated in tumors compared to normal tissues (Figure 3C,D). A heat map further revealed that interactions between SRSF1+ malignant epithelial cells and fibroblasts were particularly frequent and strong (Figure 3E), while distinct signaling patterns between cell subpopulations in normal versus cancer settings were delineated (Figure 3F). Focusing on SRSF1+ malignant epithelial cells as a signal source, Figure 3G specifically compares the outgoing ligand‐receptor signal strength to various receptor cell subpopulations in both conditions. Finally, the landscape of dysregulated communication is detailed through bubble plots showcasing the upregulated and downregulated ligand–receptor pairs in cancer (Figure 3H,I). Specifically, key protumorigenic signaling networks, including the TGF‐*β* and vascular endothelial growth factor (VEGF) pathways, were found to be significantly activated during interactions between SRSF1+ malignant epithelial cells and fibroblasts.

### 3.4. Mutational Analysis Between SRSF1‐Low and High Tumor Samples

To investigate the mutational landscape associated with SRSF1 expression in GC, we stratified TCGA‐STAD tumor samples into SRSF1‐low (bottom quartile, *n* = 102) and SRSF1‐high (top quartile, *n* = 102) groups. Oncoplots revealed distinct mutation profiles between the two groups (Figure S1A,B). In SRSF1‐low tumors, the most frequently mutated genes included TTN (48%), TP53 (42%), MUC16 (33%), FLG (30%), and SYNE1 (29%), with ARID1A mutations occurring in 27% of cases. Conversely, SRSF1‐high tumors exhibited higher mutation frequencies in TTN (57%), TP53 (47%), and MUC16 (32%), alongside elevated ARID1A (28%) and CSMD3 (26%) mutations. Pairwise co‐occurrence and mutual exclusivity analysis revealed divergent mutational patterns between the two groups (Figure S1C). Notably, ARID1A and TP53 showed significant mutual exclusivity in the SRSF1‐low group, suggesting distinct oncogenic dependencies in this molecular subtype. Forest plot analysis of differentially mutated genes identified three genes with significant enrichment: ZNF98 and NBN were preferentially mutated in SRSF1‐low tumors (OR = 0 and 0.092, respectively; both *p* < 0.01), whereas APBA2 mutations were enriched in SRSF1‐high tumors (OR = 12.099, *p* < 0.01) (Figure S1D). Lollipop plots illustrated the distribution of hotspot mutations across these genes, revealing that ZNF98 harbored missense mutations exclusively in SRSF1‐low samples, APBA2 displayed clustered missense mutations predominantly in SRSF1‐high tumors, and NBN showed missense mutations in SRSF1‐low samples with a frameshift deletion restricted to SRSF1‐high tumors (Figure S1E–G). Collectively, these findings demonstrate that SRSF1 expression levels delineate GCs with distinct mutational spectra and intertumoral heterogeneity, providing a potential framework for mutation‐ and transcriptome‐based molecular subtyping of GC.

### 3.5. SRSF1 Overexpression Promotes Invasion, Migration, and Apoptosis Resistance in GC Cells In Vitro

To investigate the functional role of SRSF1 in GC, we performed SRSF1 knockdown and overexpression in MKN28 cells. Knockdown and overexpression transfection efficiency was confirmed by Western blot (Figure 4A,B) and qRT‐PCR (Figure 4C,D). Clonogenic assays revealed that SRSF1‐overexpressing (OE‐SRSF1) cells formed larger colonies than controls. Specifically, colony formation rates increased from 3.07*%* ± 0.27*%* in empty vector controls to 4.53*%* ± 0.31*%* in OE‐SRSF1 cells (*p* = 0.003) (Figure 4E). Transwell and Matrigel assays evaluated migration and invasion, and results demonstrated that SRSF1 overexpression enhanced these capacities in GC cells, whereas knockdown attenuated them (Figure 4F,G). Annexin V/PI flow cytometry quantified apoptotic rates across groups (Figure 4H). As shown in Figure 4I, SRSF1 overexpression significantly inhibited apoptosis: total rates fell from 9.05*%* ± 0.33*%* (controls) to 6.07*%* ± 0.22*%* (*p* < 0.001), early‐stage apoptosis from 2.56*%* ± 0.25*%* to 1.13*%* ± 0.12*%* (*p* < 0.001), and late‐stage apoptosis from 6.49*%* ± 0.35*%* to 4.94*%* ± 0.21*%* (*p* < 0.01). These findings indicate that SRSF1 promotes cell survival by inhibiting apoptosis. Cell cycle analysis was performed via PI staining and flow cytometry (Figure 4J). As shown in Figure 4K, in SRSF1‐knockdown (KD‐SRSF1) versus knockdown negative control (KD‐NC) cells, the G_0_/G_1_‐phase fraction was slightly elevated (36.39*%* ± 0.44*%* vs. 34.57*%* ± 2.40*%*, *p* > 0.05), while the S‐phase fraction decreased significantly (57.68*%* ± 0.91*%* vs. 60.91*%* ± 1.59*%*, *p* < 0.05). Conversely, in OE‐SRSF1 versus overexpression negative control (OE‐NC) cells, G_0_/G_1_ fraction was increased (34.89*%* ± 2.49*%* vs. 28.80*%* ± 1.07*%*, *p* < 0.05), and the S‐phase fraction trended lower (62.88*%* ± 2.16*%* vs. 69.19*%* ± 0.94*%*, *p* < 0.05). CCK‐8 assays evaluated the impact of SRSF1 on cell viability. As shown in Figure 4L, compared to controls, SRSF1 knockdown suppressed MKN28 cell viability (*p* < 0.001 at 24 h and *p* < 0.05 at 48 h), whereas overexpression enhanced it (*p* < 0.001 at 24 h). However, the viability‐enhancing effect of overexpression was transient: No significant differences were observed at 48 h (*p* > 0.05) or for either group at 72 h (*p* > 0.05).

Figure 4Functional impacts of SRSF1 on cellular processes in MKN28 cells. (A) Western blot analysis confirmed the efficiency of SRSF1 knockdown in MKN28 cells. (B) Western blot validation of SRSF1 protein levels in the overexpression group. (C) qRT‐PCR analysis verified SRSF1 knockdown efficiency at the mRNA level. (D) qRT‐PCR analysis verified SRSF1 overexpression efficiency at the mRNA level. (E) Clonogenic assays showed that SRSF1‐overexpressing cells formed larger colonies, with colony formation rates increasing from 3.07*%* ± 0.27*%* in empty vector controls to 4.53*%* ± 0.31*%* in SRSF1‐overexpressing cells (*p* = 0.003). (F) Transwell migration assays demonstrated that SRSF1 overexpression enhanced migration capacity in GC cells, whereas knockdown attenuated it. (G) Matrigel invasion assays showed that SRSF1 overexpression enhanced invasion capacity, and knockdown reduced it. (H) Representative flow cytometry plots of Annexin V/PI staining showing apoptotic cell populations across different groups. (I) Quantification of apoptosis rates revealed that SRSF1 overexpression significantly inhibited total apoptosis, early‐stage apoptosis, and late‐stage apoptosis. (J) Representative flow cytometry histograms of PI‐stained cells for cell cycle analysis. (K) Cell cycle quantification showed that SRSF1 knockdown slightly increased G_0_/G_1_ phase fraction and significantly decreased S‐phase fraction; conversely, SRSF1 overexpression increased G_0_/G_1_ fraction and decreased S‐phase fraction. (L) CCK‐8 assays showed that SRSF1 knockdown suppressed MKN28 cell viability at 24 h (*p* < 0.001) and 48 h (*p* < 0.05), while overexpression enhanced viability at 24 h (*p* < 0.001), with no significant differences observed at 48 or 72 h for either group (*p* > 0.05). ns: no significance; ∗ indicates *p* < 0.05; ∗∗ indicates *p* < 0.01; ∗∗∗ indicates *p* < 0.001.

**Figure:**
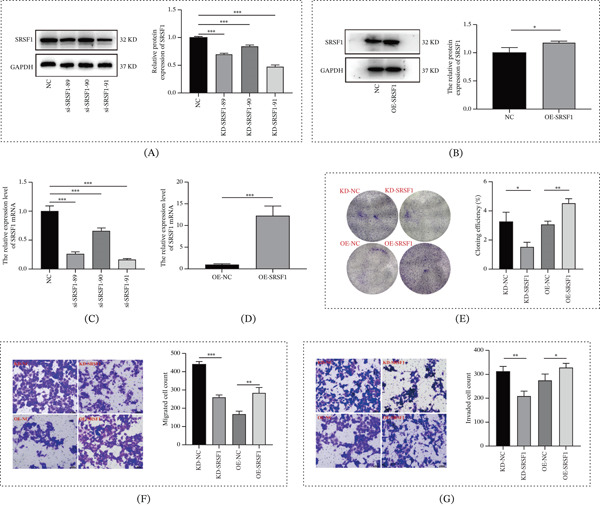
(a)

**Figure:**
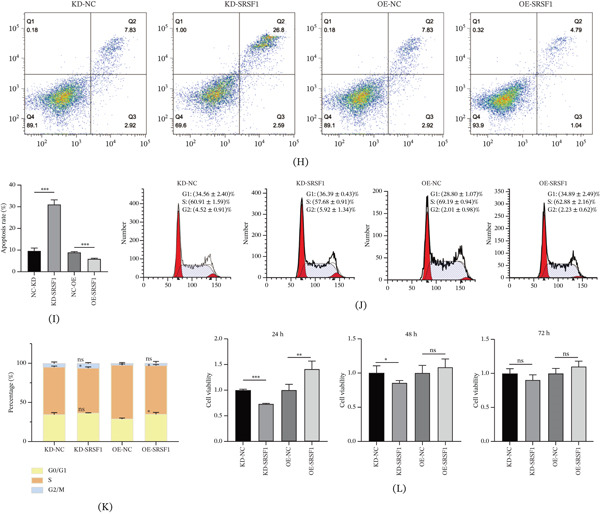
(b)

### 3.6. SRSF1 Modulates Apoptotic Protein Expression via Mcl‐1 Regulation

Prior to transfection, we measured Mcl‐1 mRNA and protein expression in normal gastric mucosal cells (GSE‐1) and GC cell lines via qRT‐PCR and Western blot. Mcl‐1 and both isoforms (Mcl‐1L/Mcl‐1S) were significantly upregulated in GC cells versus normal GSE‐1 cells at the mRNA level (Figure 5A) and the protein level (Figure 5B–D). After inducing SRSF1 overexpression/knockdown in the GC cell line MKN28 (Figure 5E), we quantified Mcl‐1 mRNA via qRT‐PCR at 24 h posttransfection (Figure 5F), and concurrently assessed protein levels of Mcl‐1 isoforms (Mcl‐1S/Mcl‐1 L) (Figure 5G–I). Subsequently, we measured downstream mitochondrial apoptosis markers (BAK, cleaved caspase‐9, and cleaved caspase‐3). qRT‐PCR analysis revealed that compared with KD‐NC, SRSF1 knockdown significantly upregulated CASP3, CASP9, and BAK1 mRNA levels (*p* < 0.05) (Figure 5J–L). Following SRSF1 overexpression, Mcl‐1 mRNA showed no significant downregulation, whereas CASP3, CASP9, and BAK1 mRNA levels were all significantly downregulated (Figure 5J–L). Collectively, these data demonstrate that SRSF1 exerts a modest negative transcriptional regulation on Mcl‐1 (not statistically significant) but significantly suppresses mitochondrial apoptosis‐related genes. This transient accumulation of Mcl‐1 mRNA following SRSF1 knockdown may result from the disruption of the alternative splicing equilibrium, a phenomenon further explored in the Discussion section. Western blot analysis revealed that SRSF1 overexpression or knockdown induced coordinated changes in Mcl‐1L and Mcl‐1S protein levels, though the regulatory effect on Mcl‐1L was not statistically significant (Figure 5G). Compared with OE‐NC, SRSF1 overexpression significantly reduced Mcl‐1S expression (1.0 ± 0.10 vs. 1.62 ± 0.19; *p* < 0.01; Figure 5I), whereas SRSF1 knockdown markedly increased Mcl‐1S protein levels (1.0 ± 0.06 vs. 0.69 ± 0.05; *p* < 0.01; Figure 5I). Collectively, these results demonstrate that SRSF1 regulates Mcl‐1 protein expression, exerting a more consistent regulatory effect on Mcl‐1S than on Mcl‐1L. Furthermore, we performed Western blot analysis to assess the protein levels of BAK1, cleaved caspase‐3, and cleaved caspase‐9 when SRSF1 was knocked down or overexpressed (Figure 5M). We observed downregulated mitochondrial apoptosis effectors versus controls: BAK1 (0.52 ± 0.08 vs. 1.0 ± 0.04, *p* < 0.01; Figure 5N), cleaved caspase‐3 (0.55 ± 0.10 vs. 1.0 ± 0.07, *p* < 0.01; Figure 5O), and cleaved caspase‐9 (0.63 ± 0.07 vs. 1.0 ± 0.07, *p* < 0.01; Figure 5P). Conversely, SRSF1 overexpression exhibits an opposite trend. Given Mcl‐1’s well‐documented role as an upstream regulator of mitochondrial apoptosis [15], our data support the hypothesis that SRSF1 mediates this pathway via transcriptional and posttranslational control of Mcl‐1. To explore the role of SRSF1 in tumor immunoregulation, we analyzed RNA‐seq data and identified significant correlations between SRSF1 expression and immune cell infiltration density. The Spearman correlation analysis revealed that higher SRSF1 expression was inversely associated with resting mast cells (rho = −0.32, *p* < 0.001), monocytes (rho = −0.38, *p* < 0.001), activated NK cells (rho = −0.38, *p* < 0.001), and regulatory T cells (Tregs; rho = −0.34, *p* < 0.05)—but positively correlated with resting NK cells (rho = 0.32, *p* < 0.001), activated memory CD4^+^ T cells (rho = 0.31, *p* < 0.001), follicular helper T (Tfh) cells (rho = 0.30, *p* < 0.001), and *γδ* T cells (rho = 0.20, *p* < 0.01; all *p* values FDR‐corrected) **(**Figure 5Q**)**. These findings suggest that SRSF1 expression correlates with estimated impaired innate immune cell infiltration (e.g., NK cell activation) and suppressed Treg infiltration (TIMER). However, increased frequencies of specific T cell subsets observed in our results suggest immune dysregulation—a phenomenon potentially driven by genomic instability.

Figure 5Regulation of MCL‐1expression and its functional role in GC cells. (A) qRT‐PCR showed that Mcl‐1 mRNA, including both Mcl‐1L and Mcl‐1S isoforms, was significantly upregulated in gastric cancer (GC) cell lines compared with normal GSE‐1 cells. (B) Western blot confirmed that Mcl‐1 protein levels were elevated in GC cell lines versus GSE‐1 cells. (C) Quantification of Mcl‐1L protein isoform showed significant upregulation in GC cells. (D) Quantification of Mcl‐1S protein isoform also showed significant upregulation in GC cells. (E) Western blot confirmed successful SRSF1 overexpression and knockdown in MKN28 cells for subsequent experiments. (F) qRT‐PCR at 24 h posttransfection revealed no significant change in Mcl‐1 mRNA following SRSF1 overexpression or knockdown. (G) Western blot showed that SRSF1 overexpression or knockdown induced coordinated changes in Mcl‐1L and Mcl‐1S protein levels. (H) Quantification of Mcl‐1L protein showed no statistically significant regulation by SRSF1. (I) Quantification of Mcl‐1S protein demonstrated that SRSF1 overexpression significantly reduced Mcl‐1S expression. (J–L) qRT‐PCR showed that SRSF1 knockdown significantly upregulated CASP3 mRNA (*p* < 0.05), whereas SRSF1 overexpression significantly downregulated it. Similar results were observed for CASP9 mRNA, with knockdown increasing and overexpression decreasing its levels (*p* < 0.05); BAK1 mRNA was also significantly upregulated upon SRSF1 knockdown and downregulated upon overexpression (*p* < 0.05). (M) Western blot assessed protein levels of mitochondrial apoptosis effectors (BAK1, cleaved caspase‐3, and cleaved caspase‐9) following SRSF1 modulation. (N) SRSF1 knockdown significantly increased BAK1 protein compared with controls (*p* < 0.01), while overexpression showed an opposite trend. (O) Cleaved caspase‐3 protein was significantly elevated after SRSF1 knockdown (*p* < 0.01) and reduced after overexpression. (P) Cleaved caspase‐9 protein followed the same pattern, with significant upregulation upon knockdown (*p* < 0.01). (Q) Spearman correlation analysis of RNA‐seq data revealed that SRSF1 expression was inversely correlated with resting mast cells, monocytes, activated NK cells, and regulatory T cells, but positively correlated with resting NK cells, activated memory CD4^+^ T cells, follicular helper T cells, and *γδ* T cells. ns: no significance; ∗ indicates *p* < 0.05;  ^∗∗^ indicates *p* < 0.01; ∗∗∗ indicates *p* < 0.001.

**Figure:**
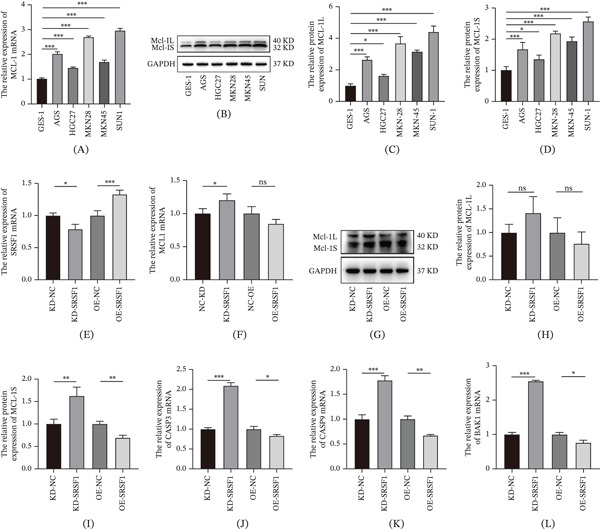
(a)

**Figure:**
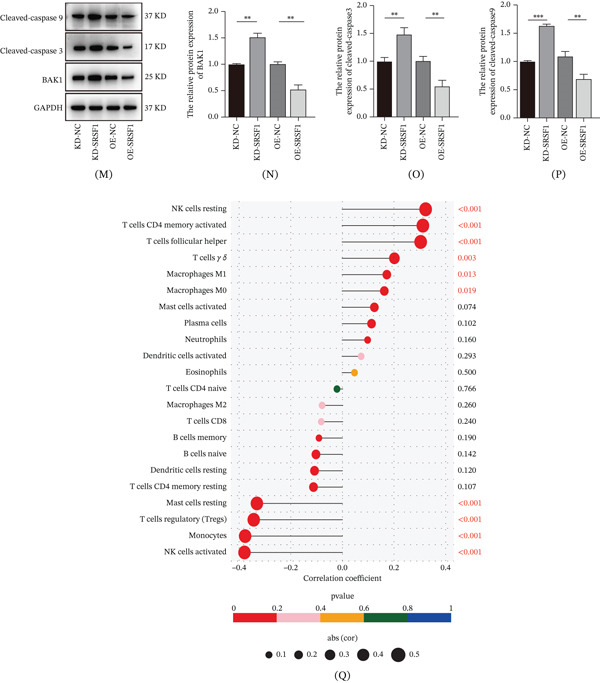
(b)

### 3.7. Evaluating Impact of SRSF1 on Tumor Growth and Apoptosis‐Related Proteins in Animal Models

**Figure 6:**
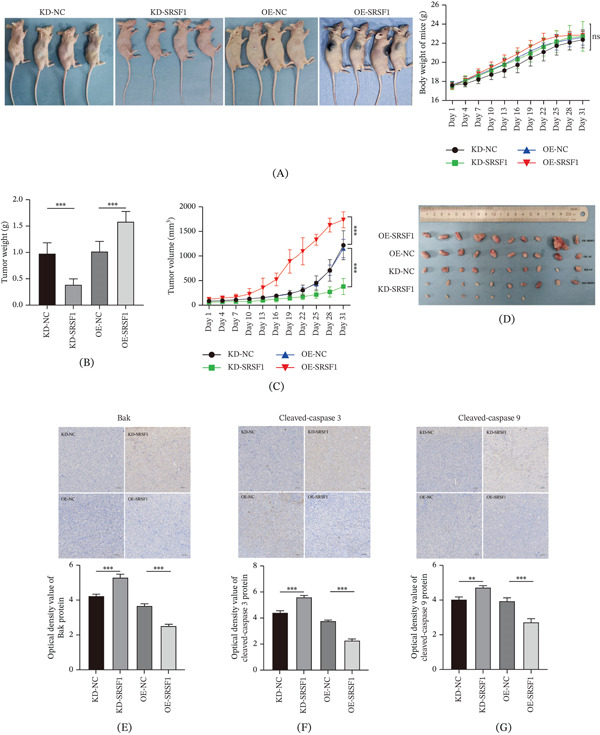
Effects of SRSF1 on tumor growth and apoptosis‐related protein expression in vivo. (A) The animals were divided into four treatment groups, and body weight measurements throughout the observation period showed no significant differences among groups, confirming that physiological variations did not confound subsequent in vivo tumor analyses (figure is a representative set of images). (B) SRSF1 overexpression significantly increased tumor weight by approximately 1.6‐fold compared with controls (*p* < 0.001), while SRSF1 knockdown reduced tumor weight to 60% of control levels (*p* < 0.001). (C) Tumor volume followed a similar trend: overexpression expanded volume by about 1.5‐fold (*p* < 0.001), whereas knockdown reduced volume to 31% of controls (*p* < 0.001). (D) Gross appearance of dissected tumor specimens from control, SRSF1‐overexpressing (OE), and SRSF1‐knockdown (KD) groups. (E) Immunohistochemical quantification showed that Bak expression was reduced to 68% of control levels in OE tumors (*p* < 0.001) and increased 1.25‐fold in KD tumors (*p* < 0.001). (F) Cleaved caspase‐3 expression was reduced by approximately 60% in OE tumors (*p* < 0.001) and increased 1.27‐fold in KD tumors (*p* <0.001). (G) Cleaved caspase‐9 expression decreased by about 69% in OE tumors (*p* <0.001) and increased 1.2‐fold in KD tumors (*p* < 0.001). ns: no significance; ∗ indicates *p* < 0.05; ∗∗ indicates *p* < 0.01; ∗∗∗ indicates *p* < 0.001.

We established murine tumor‐bearing models and measured tumor weight/volume postimplantation. Immunohistochemistry detected apoptosis‐associated proteins—Bak, cleaved caspase‐9, and cleaved caspase‐3—in tumor tissues. Body weight showed no intergroup differences throughout observation (one‐way ANOVA, *p* > 0.05), confirming that physiological variations did not confound subsequent in vivo tumor analyses (Figure 6A). SRSF1 overexpression accelerated tumor progression: compared to controls, the OE group tumor weight increased ~1.6‐fold (control: 1.01 ± 0.19 g; OE: 1.58 ± 0.19 g; *p* < 0.001), while the KD group weight was reduced to 60% of controls (control: 0.97 ± 0.20 g; KD: 0.39 ± 0.11 g; *p* < 0.001; Figure 6B). Tumor volume followed a similar trend: OE volume expanded ~1.5‐fold (control: 1152.21 ± 185.9 mm^3^; OE: 1735.9 ± 161.2 mm^3^; *p* < 0.001), whereas KD volume was diminished to 31% of controls (KD: 383.6 ± 164.6 mm^3^ vs. control: 1221.4 ± 294.2 mm^3^; *p* < 0.001; Figure 6C). The gross appearance of the dissected tumor specimen is shown in Figure 6D. These data collectively demonstrate that SRSF1 plays an intrinsic protumorigenic role in vivo. IHC staining revealed significantly reduced expression of proapoptotic regulators—Bak, cleaved caspase‐9, and cleaved caspase‐3—in OE‐SRSF1 tumors versus controls. Quantitative analysis showed that Bak expression was reduced to 68% of control levels in OE tumors (OD: 2.53 ± 0.09 vs. 3.68 ± 0.12; *p* < 0.001) and increased 1.25‐fold in KD tumors (4.24 ± 0.11; *p* < 0.001; Figure 6E). Cleaved caspase‐3 expression was reduced by ~60% in OE tumors (OD: 2.27 ± 0.11 vs. 3.77 ± 0.07; *p* < 0.001), whereas it increased 1.27‐fold in KD tumors (5.61 ± 0.12; *p* < 0.001; Figure 6F). Cleaved caspase‐9 expression decreased by ~69% in OE tumors (OD: 2.72 ± 0.20 vs. 3.94 ± 0.19; *p* < 0.001) and increased 1.2‐fold in KD tumors (4.73 ± 0.09; *p* < 0.001; Figure 6G). These findings confirm that SRSF1 promotes in vivo tumorigenesis by enhancing tumor growth and inhibiting apoptosis.

## 4. Discussion

GC ranks fifth globally in both incidence and mortality among malignant neoplasms, with a pathogenesis that remains poorly understood. In recent years, the role of alternative splicing in malignant tumor development and progression has garnered significant research attention. Bioinformatic analyses confirmed upregulation of SRSF1 in multiple malignancies, including GC, suggesting a procarcinogenic role. Subsequent correlation analyses with clinical features showed no association with TNM stage but significant links to age (> 65 years) and gender. In vivo and in vitro assays confirmed that SRSF1 overexpression significantly enhanced migration, invasion, and antiapoptotic activity in GC cells. However, corresponding assays showed no significant increases in cell viability or cell cycle progression. This indicates that SRSF1 primarily drives GC progression via metastatic processes rather than proliferation. Crucially, genetic modulation of SRSF1 (overexpression or knockdown) revealed its regulatory effects on both the transcriptional and protein levels of the apoptosis regulator Mcl‐1. Specifically, quantitative assays showed that SRSF1 suppresses the expression of the proapoptotic Mcl‐1S isoform. Our findings are highly consistent with studies in other cancers, where SRSF1 shifts the splicing balance toward the antiapoptotic Mcl‐1L isoform. By selectively suppressing the proapoptotic Mcl‐1S (which lacks the BH3 domain), SRSF1 shifts the overall BCL‐2 family balance. This prevents the oligomerization of BAX and BAK, thereby sealing the mitochondrial outer membrane and effectively shutting down the caspase‐9/caspase‐3 apoptotic cascade. Subsequent analysis of Mcl‐1 downstream apoptosis targets—including cleaved caspase‐9, cleaved caspase‐3, and Bak—revealed that SRSF1 overexpression significantly reduced the transcriptional levels and protein translation of proapoptotic effectors. Quantification further showed a strong negative correlation between SRSF1 levels and these apoptosis execution markers. Separately, analysis of immune infiltration in the TME revealed a clear correlation between SRSF1 expression and immunosuppressive cellular profiles, establishing upregulated SRSF1 as a hallmark of T cell–dysregulated niches. Collectively, these findings provide novel insights into SRSF1‐mediated epigenetic reprogramming during gastric carcinogenesis.

Additionally, our bioinformatic analysis further expands on the regulatory network of SRSF1 in GC from single‐cell, interactome, and genomic perspectives. Single‐cell RNA sequencing reveals that SRSF1‐positive malignant epithelial cells exhibit unique transcriptomic characteristics within the TME. Their coexpression network modules, such as M4, strongly correlate with SRSF1 expression and are enriched in apoptosis‐inhibition and immune‐regulation pathways. This finding is consistent with in vitro experiments, indicating that SRSF1 shapes the antiapoptotic phenotype of cell subpopulations by regulating gene splicing preferences, such as the suppression of Mcl‐1S. Cell interaction analysis reveals enhanced signaling between SRSF1‐positive cells and fibroblasts, particularly in the TGF‐*β* and VEGF pathways, suggesting that SRSF1 may indirectly drive invasion by remodeling stromal interactions in the TME, such as promoting fibroblast activation. Alterations in this interaction pattern may synergize with the SRSF1‐mediated immunosuppressive microenvironment, reducing Treg cell infiltration and accelerating disease progression. Mutational profiling, at the genomic level, confirms that tumors with high SRSF1 expression are enriched for mutations in genes such as APBA2. These mutations may create a positive feedback loop with SRSF1’s splicing functions, affecting DNA repair pathways and thereby promoting tumor heterogeneity and chemotherapy resistance. In summary, multidimensional bioinformatic data validate the role of SRSF1 as a core regulatory factor in GC, driving immune evasion, metabolic reprogramming, and genomic instability through integrated variations in the transcriptome (single‐cell modules), interactome (cell interaction), and genome (mutational profile). This provides theoretical grounding for combined therapies targeting the SRSF1‐Mcl‐1 axis, such as immune checkpoint inhibitors combined with splicing modulators.

Furthermore, our bioinformatic analysis reveals that SRSF1‐positive malignant epithelial cells exhibit unique transcriptomic characteristics. Cell interaction analysis shows enhanced signaling pathways between SRSF1‐positive cells and fibroblasts, particularly in TGF‐*β* and VEGF pathways, suggesting that SRSF1 may indirectly drive invasion by remodeling stromal interactions in the TME. Mutational profiling confirms that tumors with high SRSF1 expression are enriched with specific mutations, potentially creating a positive feedback loop with SRSF1’s splicing functions. SRSF1 has predominantly been studied as a downstream target rather than a regulatory driver in prior GC research. For instance, OnclncRNA‐626 stabilizes SRSF1 via direct binding, thereby inactivating the p53 pathway and accelerating malignant progression [16]. Similarly, HOXA11‐AS promotes GC cell proliferation and invasion through SRSF1‐dependent mechanisms [17]. Nevertheless, the precise regulatory mechanisms of SRSF1 in GC—and its functional crosstalk with core signaling cascades—remain poorly characterized. This study is the first to directly investigate SRSF1 as a primary regulatory driver of GC pathogenesis. Using integrated in vitro assays and in vivo transplantation models, we establish SRSF1 as a critical accelerator of GC progression. Functional analyses reveal that SRSF1 overexpression markedly enhances malignant phenotypes, significantly increasing invasive capacity and migratory potential in GC cell lines. This pro‐oncogenic function aligns directly with SRSF1’s established role as a canonical tumor‐promoting factor that drives cellular motility and metastatic competence. Analysis of effector protein expression in SRSF1‐modified GC cell cohorts revealed distinct alterations in key proapoptotic mediators (BAK, cleaved caspase‐9, and cleaved caspase‐3), demonstrating that SRSF1 facilitates tumor progression by conferring enhanced antiapoptotic properties to GC cells. However, cell viability assays showed a transient proliferative advantage at 24 h postinduction, which dissipated completely by 48–72 h, indicating that SRSF1 overexpression does not drive sustained or potent cell proliferation.

Analysis of GC cell cycle dynamics revealed paradoxical findings: OE‐SRSF1 populations exhibited significantly higher G_0_/G_1_ phase occupancy, whereas KD‐SRSF1 populations showed modest increases that were statistically indistinguishable from controls. Notably, both experimental groups showed concurrent S‐phase depletion compared to controls, leading us to hypothesize that this outcome may stem from the complexity of biological mechanisms—such as the multitarget regulatory effects of SRSF1 on splicing events. As a splicing factor, SRSF1 regulates splicing events of hundreds to thousands of genes, and its impact on the cell cycle arises from multitarget, multipathway actions—manifested as concurrent changes in S‐phase and G_0_/G_1_‐phase populations. This likely stems from SRSF1 simultaneously suppressing splice events that promote S phase (e.g., splicing of DNA replication‐related genes) and those that drive exit from G_0_/G_1_, resulting in fewer S‐phase cells and an accumulation of G_0_/G_1_‐phase cells. However, temporal analysis resolves this apparent discrepancy: the early proliferative advantage contrasts with the antiproliferative phenotype, suggesting that OE‐SRSF1 cells redirect resources to motility, invasion, and activation of survival pathways rather than sustained mitotic progression. Multiple cancer models exhibit the “proliferation‐migration paradox”—a phenomenon where highly migratory cells display reduced proliferation rates [18]. Our findings suggest that SRSF1 overexpression may shift cells toward a migration‐prioritized phenotype, aligning with the well‐documented proliferation‐migration trade‐off. Concurrently, the transient proliferation advantage observed at the 24‐h timepoint in CCK‐8 assays aligns temporally with subtle cellular dynamic changes during cell cycle progression. This early‐phase advantage likely stems from two interrelated mechanisms: (i) Cell cycle desynchronization: Viral transduction inherently induces partial cellular synchronization; SRSF1 overexpression accelerates the cell cycle transition of these synchronized cells, producing a detectable proliferation differential in early monitoring periods. (ii) Transient mitogenic signaling: SRSF1 triggers an immediate growth‐promoting signal that is subsequently overridden or counteracted by intrinsic feedback—stemming from activated migratory/invasive programs and metabolic resource reallocation—ultimately attenuating sustained proliferation. Cell cycle results showed a significant increase in the proportion of cells in the G_0_/G_1_ phase in the OE‐SRSF1 group, which can be linked to the migratory phenotype—sometimes, to acquire migratory capacity, cells enter a relatively quiescent or reversible cell cycle arrest state (similar to the G_0_/G_1_ phase) to reshape their cytoskeleton and adhesion structures in preparation for migration [19]. Therefore, the core function of SRSF1 is to promote tumor invasion, with its most prominent phenotype being enhanced cell migration and invasion capacity; its direct impact on proliferation is likely secondary and transient. It may fine‐tune proliferation by regulating a specific set of splicing targets (e.g., cell cycle checkpoint‐related genes), rather than directly and powerfully driving the cell cycle engine like classic oncogenes (e.g., Myc). This aligns with Fu et al.’s findings in GC, where SRSF1 drives GC cell migration, invasion, and metastasis by regulating epithelial–mesenchymal transition (EMT) [20]. Similarly, in other tumors [21], SRSF1 overexpression has also been shown to induce the EMT phenotype.

Mcl‐1, an antiapoptotic protein of the Bcl‐2 family, plays a critical role in medicine—particularly in tumor research [22]. It inhibits apoptosis by binding to proapoptotic Bcl‐2 family proteins, thereby regulating cell survival and apoptosis [23, 24]. Aberrant Mcl‐1 expression or gene amplification is common in multiple human malignancies and closely linked to tumorigenesis, poor prognosis, and treatment resistance [6, 25]. Its distinct splicing isoforms, Mcl‐1L and Mcl‐1S, exhibit divergent mechanistic roles in apoptosis: Mcl‐1L primarily functions as an antiapoptotic protein, whereas Mcl‐1S displays proapoptotic activity [26–28]. Mcl‐1L, the long isoform of the Bcl‐2 family antiapoptotic protein Mcl‐1, contains BH1, BH2, and BH3 domains [26]. It exerts antiapoptotic effects by binding to proapoptotic Bcl‐2 family members (e.g., BAX and BAK), thereby preventing their oligomerization. This blocks mitochondrial outer membrane permeabilization, inhibits cytochrome c release, and suppresses downstream caspase cascade activation and apoptosis [28, 29]. Mcl‐1S is a proapoptotic isoform of the Bcl‐2 family member Mcl‐1 generated via alternative splicing; its structure undergoes a frameshift due to skipping of Exon 2, resulting in retention of only the BH3 domain, and it functions oppositely to the antiapoptotic isoform Mcl‐1L, which harbors BH1, BH2, BH3, and transmembrane domain [15]. Previous studies have demonstrated that in breast cancer, choriocarcinoma, and esophageal squamous cell carcinoma, the expression of Mcl‐1S is regulated by the splicing factor SRSF1: SRSF1 binds to specific sequences (e.g., the GAAGAA motif) in Mcl‐1 pre‐mRNA, promotes retention of Exon 2, thereby increasing Mcl‐1L production and suppressing Mcl‐1S, and ultimately enhances tumor cell survival [15, 30]. In our prior studies, we demonstrated that Mcl‐1 is overexpressed in GC tissues, and this overexpression correlates with poor prognosis in patients. Its expression levels are associated with pathological features—including TNM stage, lymph node metastasis, and histological differentiation—and imbalanced splicing of Mcl‐1 isoforms further links to the proliferative, migratory, and invasive phenotypes of GC. Splicing dysregulation is a recognized core hallmark of tumorigenesis and cancer progression, driving apoptosis resistance [9]. In cancer research—especially for GC—aberrant Mcl‐1 expression and splicing imbalance are major contributors to tumor development, progression, and therapeutic resistance. Therefore, targeting Mcl‐1L or modulating the Mcl‐1L/Mcl‐1S ratio has emerged as a research focus and key strategy for developing novel anticancer therapies.

Serine/arginine‐rich (SR) proteins—key RNA‐binding proteins—regulate exon alternative splicing by binding specifically to splicing enhancer elements (ESEs) in pre‐mRNA and cooperating with spliceosomal core components (e.g., U1 snRNP and U2AF). Additionally, their function is tightly controlled by posttranslational modifications (PTMs), such as phosphorylation mediated by SRPK and CLK kinases, which modulate their nuclear‐cytoplasmic distribution and their ability to govern specific splicing events [31]. Thus, SR proteins not only directly fine‐tune RNA processing via alternative splicing but also bridge environmental cues to gene regulation through protein–protein interactions and signal transduction. SRSF1, a key member of the SR protein family, plays a critical role in maintaining the balance between cellular survival and apoptosis by promoting the prosurvival Mcl‐1L isoform and suppressing the proapoptotic Mcl‐1S isoform. This regulatory function of SRSF1 depends on its binding affinity for Mcl‐1 mRNA and its PTM status, enabling adaptive regulation in pathological contexts such as cancer. Dysregulated SRSF1 expression or function drives aberrant Mcl‐1 splicing, conferring a survival advantage to tumor cells and contributing to tumorigenesis and drug resistance [15, 31, 32]. These findings demonstrate that SR proteins—particularly SRSF1—exert multilayered, complex regulation of alternative splicing at both the RNA and protein levels, while also representing potential therapeutic targets.

In this study, we assessed changes in total Mcl‐1 mRNA levels and the protein expression of its two splice isoforms (Mcl‐1L and Mcl‐1S) by either overexpressing or knocking down SRSF1. Results demonstrated that alterations in SRSF1 expression exerted significant but asymmetric effects on total Mcl‐1 mRNA: SRSF1 knockdown significantly upregulated Mcl‐1 mRNA (*p* < 0.05), whereas SRSF1 overexpression only induced a mild downregulation (*p* > 0.05). SRSF1 primarily modulates the generation of specific Mcl‐1 splice isoforms (Mcl‐1L and Mcl‐1S) via splicing regulation, suggesting it likely does not directly affect the overall mRNA transcription rate of the Mcl‐1gene [31]. Consistent with this, SRSF1 overexpression failed to significantly reduce total mRNA levels, while knockdown disrupted the normal regulatory balance more sensitively, thereby significantly impacting mRNA accumulation.

At the protein level, Mcl‐1S is subject to significant negative regulation by SRSF1: Knockdown of SRSF1 led to a marked increase in Mcl‐1S protein levels (*p* < 0.01), whereas its overexpression led to a significant decrease (*p* < 0.01). Notably, although Mcl‐1L showed a regulatory trend similar to Mcl‐1S (mild upregulation upon SRSF1 knockdown and mild downregulation upon overexpression), this difference did not reach statistical significance (*p* > 0.05). These results highlight the central role of SRSF1 in splicing regulation—particularly its selective inhibitory control over the proapoptotic isoform Mcl‐1S. This aligns with previous studies, which showed that SRSF1 selectively recognizes ESEs in Mcl‐1 mRNA, preferentially suppressing Mcl‐1S production while exerting weaker modulation on Mcl‐1L. This phenomenon may be attributed to the presence of high‐affinity SRSF1 binding sites in the exon–intron junction region of the Mcl‐1S isoform [33]. Furthermore, the binding affinity between spliceosomal core components (e.g., U1 snRNP and U2AF) and SR proteins directly dictates splicing efficiency. Studies have demonstrated that SRSF1, by coordinating with these components, selectively regulates Mcl‐1S production more efficiently, thereby exerting a more pronounced negative effect on Mcl‐1S [32]. Our future studies will further investigate the binding sites of SRSF1 with Mcl‐1 pre‐mRNA, spliceosome assembly mechanisms, and the translation/protein degradation kinetics of Mcl‐1L/Mcl‐1S mRNAs in GC cell—to comprehensively elucidate the fine‐tuning regulatory mechanisms underlying SRSF1’s modulation of Mcl‐1 isoform expression in GC. Furthermore, IHC analysis of experimental cells and tumor‐bearing xenografts revealed that SRSF1 expression negatively regulates downstream apoptotic proteins of Mcl‐1 (e.g., C‐Caspase9, C‐Caspase3, and Bak). Thus, we hypothesize that SRSF1 regulates Mcl‐1 expression to define the antiapoptotic phenotype of GC cells.

Immune cell‐infiltrated tumor microenvironments (TIME) play a critical role in tumor research: They not only shape tumor initiation, progression, metastasis, and therapeutic response but also are a major focus of current tumor immunotherapy efforts [34], The TME comprises a diverse array of immune cells, including T cells, B cells, macrophages, dendritic cells, NK cells, and myeloid‐derived suppressor cells (MDSCs) [35, 36]. The infiltration levels and functional states of these immune cells in tumors profoundly affect patient prognosis and immunotherapy outcomes. We leveraged the TIMER database to assess correlations between SRSF1 expression and both TME immune cell infiltration levels and tumor mutation burden (TMB), revealing complex associations. SRSF1 was significantly negatively correlated with resting mast cells, monocytes, activated NK cells, and Tregs—suggesting it may promote tumor immune escape by suppressing the activity or infiltration of innate and adaptive immune cells. This is consistent with findings by Wahid et al., who reported that SRSF1 may attenuate Treg immunosuppressive function and suppress NK cell cytotoxicity—either by regulating immune checkpoint molecule expression (e.g., alternative splicing of PD‐1) or by secreting inhibitory factors (e.g., TGF‐*β* and IL‐10) [37]. Conversely, SRSF1 was positively correlated with resting NK cells, TMB, activated CD4^+^ memory T cells, Tfh cells, and *γδ* T cells—suggesting its potential to activate or maintain specific immune cell subsets. This is consistent with findings by Qi et al. that SRSF1 may enhance the antigen‐presenting capacity of CD4^+^ memory T cells by regulating Type I interferon signaling (e.g., IRF7 and IL27ra) [38]. Taken together, SRSF1—via multidimensional regulation of immune cell function and tumor‐intrinsic metabolism—may serve as a key target with dual potential for prognostic assessment and therapeutic intervention in cancer immunotherapy.

In our animal experiments, tumor‐bearing models revealed that OE‐SRSF1 tumors grew significantly larger than OE‐NC tumors, whereas KD‐SRSF1 markedly inhibited tumor growth. This contrasts with our in vitro findings on cell proliferation and cell cycle—yet it implies that SRSF1 may drive tumor progression via nonproliferative mechanisms (e.g., immune evasion or metabolic reprogramming). Based on our single‐cell interaction analyses, we propose that the TME serves as the central mediator that translates SRSF1 expression into robust in vivo tumor growth. We hypothesize that SRSF1 drives tumorigenesis via a TME‐centric mechanism: (1) Immune‐suppressive TME remodeling: SRSF1 may reshape the immune‐suppressive microenvironment by enhancing crosstalk with cancer‐associated fibroblasts (as evidenced by our scRNA‐seq data and TGF‐*β*/VEGF pathway activation) and correlating with immune evasion signatures (such as estimated increased Tregs and impaired NK cells). (2) Metabolic reprogramming and angiogenesis. (3) Cell cycle‐independent pro‐survival signaling.

Translating the SRSF1/Mcl‐1 axis into clinical practice presents both opportunities and challenges. While direct small‐molecule inhibitors of SRSF1 are scarce, upstream kinases such as SRPK1 (which phosphorylates and activates SRSF1) are targetable, with inhibitors currently under preclinical investigation. Conversely, specific Mcl‐1 inhibitors (e.g., S63845 and AMG‐176) have entered Phase I/II clinical trials for hematological malignancies. However, their application in solid tumors like GC remains challenging due to potential dose‐limiting cardiac toxicity. Future strategies may require localized delivery systems or combination therapies with immune checkpoint inhibitors to widen the therapeutic window.

However, this study has certain limitations. First, while our data strongly indicate that SRSF1 regulates Mcl‐1 splicing, direct mechanistic evidence demonstrating the physical binding of SRSF1 to specific cis‐elements on the Mcl‐1 pre‐mRNA (e.g., via RNA immunoprecipitation [RIP] or CLIP assays) is currently lacking. Future studies will prioritize these approaches to map the exact binding motifs. Second, our immune microenvironment profiling relies largely on database estimates (e.g., TIMER) and transcriptomic correlations. Direct functional validation of immune cell evasion, such as coculture assays of SRSF1‐modified GC cells with primary NK or T cells, is required to definitively prove these immunomodulatory effects.

In summary, SRSF1 drives tumor growth through multiple mechanisms, including immune suppression, metabolic reprogramming, and regulation of apoptosis. Its in vivo protumorigenic effects appear to rely more on immune evasion and metabolic adaptation within the TME than on simple cell cycle acceleration.

## 5. Conclusion

This study systematically elucidates the protumorigenic mechanism of SRSF1 in GC: SRSF1 modulates Mcl‐1 splicing to suppress the proapoptotic Mcl‐1S isoform, which in turn inhibits the mitochondrial apoptosis pathway (i.e., the Bak/caspase‐9/caspase‐3 axis) and enhances the antiapoptotic capacity of GC cells. Notably, SRSF1 exerted only a transient and limited proproliferative effect on GC cells; instead, its more central role was to significantly promote tumor migration and invasion. This protumorigenic phenotype may be attributable to an SRSF1‐mediated “motility‐proliferation priority” strategy, in which cellular resources are preferentially allocated to invasion‐related pathways—such as the alternative splicing regulation of EMT‐associated genes—rather than to sustained proliferation. Additionally, SRSF1 constructs a multidimensional protumorigenic network by reshaping the immune‐suppressive microenvironment—upregulating Tregs and MDSCs, inhibiting CD8^+^ T cell function—and driving metabolic reprogramming—enhancing glycolysis, elevating VEGF—which underscores that its potential as a GC therapeutic target requires integrating immune modulation and metabolic intervention strategies. In summary, our in‐depth characterization of SRSF1’s mechanisms in GC is expected to provide new theoretical foundations and actionable strategies for disease diagnosis, prognosis assessment, and targeted therapy development.

## Author Contributions

Xingguang Liu and Hui Cai designed the study. Xingguang liu, Deming Liu, and Shuo Liu performed the in vitro experiments. Xingguang liu and Guangming Zhang performed the in vivo experiments. Xingguang liu and Deming Liu analyzed the data and wrote the manuscript. Hui Cai supervised the study.

## Funding

The study was funded by National Natural Science Foundation of China (10.13039/501100001809) (82360498), Gansu Joint Scientific Research Fund Major Project (No. 23JRRA1537), 2025 Central‐Guided Local Science and Technology Development Fund (No. 25ZYJA003), Gansu Provincial Health Industry Science and Technology Inno (No. GSWSZD2024‐01), Gansu Province Key Talent Project (No. 2025RCXM067), and Gansu Key Laboratory of Molecular Diagnostics and Precision Medicine for Surgical Oncology (Grant 18JR2RA033).

## Ethics Statement

All animal procedures were conducted in strict accordance with the ARRIVE Guidelines 2.0 [39] (National Centre for the Replacement, Refinement and Reduction of Animals in Research [NC3Rs], 2019) and the Guidelines for the Care and Use of Laboratory Animals (National Academies of Sciences, Engineering, and Medicine, 2011). The study protocol was reviewed and approved by the Ethics Review Committee of Gansu Provincial People’s Hospital (Approval No.: 2025‐411; Approval Date: May 27, 2025).

## Consent

The authors have nothing to report.

## Conflicts of Interest

The authors declare no conflicts of interest.

## Supporting Information

Additional supporting information can be found online in the Supporting Information section.

## Supporting information


**Supporting Information 1.** Figure S1: Mutational analysis of SRSF1‐low and SRSF1‐high gastric cancer samples. (A, B) Oncoplots depict the mutational landscape in (A) SRSF1‐low (*n* = 102) and (B) SRSF1‐high (*n* = 102). TCGA‐STAD tumor samples, with top mutated genes and their frequencies indicated. (C) Co‐occurrence and mutual exclusivity analysis between the two groups, with the diagonal indicating SRSF1‐low specific (upper triangle) and SRSF1‐high specific (lower triangle) patterns; asterisks denote statistical significance. (D) Forest plot showing odds ratios (OR) with 95% confidence intervals for differentially mutated genes between SRSF1‐high and SRSF1‐low groups; dashed line indicates no effect (OR = 1). (E–G) Lollipop plots illustrating the distribution of hotspot mutations in (E) ZNF98, (F) APBA2, and (G) NBN across SRSF1‐low and SRSF1‐high samples.

**Supporting Information 2.** Table S1: shRNA sequences targeting different regions of SRSF1 coding sequence (CDS). Table S2: qRT‐PCR primer pairs targeting multiple genes (forward and reverse sequences).

## Data Availability

The data and materials supporting the findings of this study are available within the article and its Supporting Information files, or from the corresponding author upon reasonable request.
