# Supplementary figures and images for: SRSF1/Mcl‐1 Axis Drives Apoptosis Evasion and Shapes the Immune Microenvironment to Promote Gastric Cancer Progression
Source: Hum Mutat. 2026 Jun 10;2026:9554600. doi: 10.1155/humu/9554600 (PMC13254220; doi:10.1155/humu/9554600)

**A**

Altered in 91 (89.22%) of 102 samples.

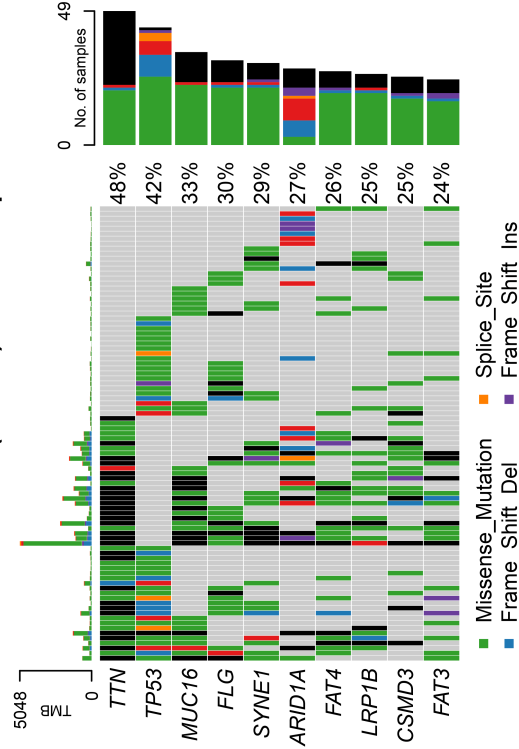**B**

Altered in 95 (93.14%) of 102 samples.

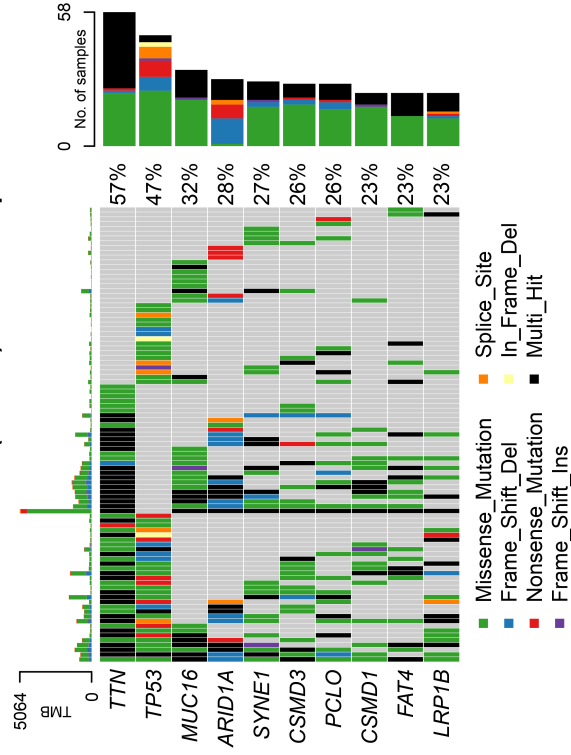**C**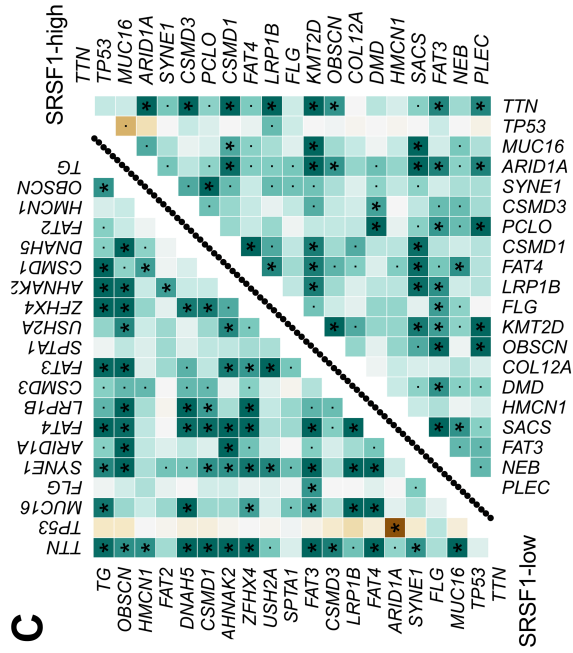**D**

High (n = 102) v/s Low (n = 102)

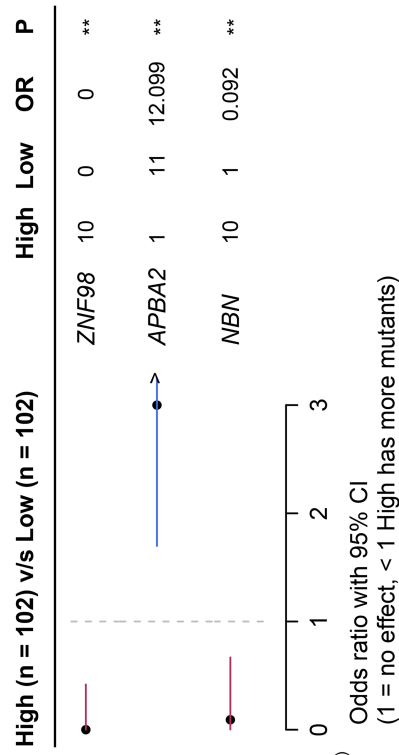**E**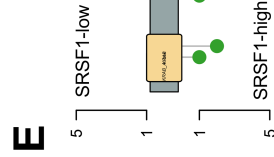**F**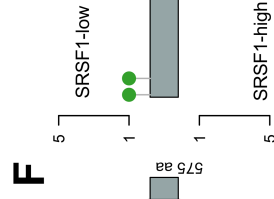**G**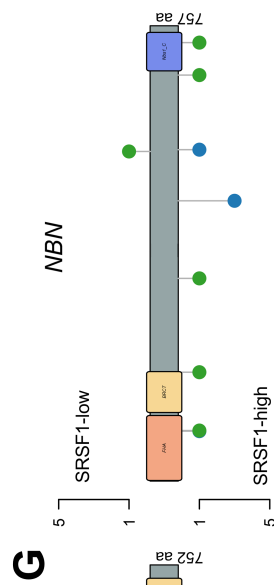

Supplement: Supplementary file 1 — Supporting Information 1. Figure S1: Mutational analysis of SRSF1‐low and SRSF1‐high gastric cancer samples. (A, B) Oncoplots depict the mutational landscape in (A) SRSF1‐low (n = 102) and (B) SRSF1‐high (n = 102). TCGA‐STAD tumor samples, with top mutated genes and their frequencies indicated. (C) Co‐occurrence and mutual exclusivity analysis between the two groups, with the diagonal indicating SRSF1‐low specific (upper triangle) and SRSF1‐high specific (lower triangle) patterns; asterisks denote statistical significance. (D) Forest plot showing odds ratios (OR) with 95% confidence intervals for differentially mutated genes between SRSF1‐high and SRSF1‐low groups; dashed line indicates no effect (OR = 1). (E–G) Lollipop plots illustrating the distribution of hotspot mutations in (E) ZNF98, (F) APBA2, and (G) NBN across SRSF1‐low and SRSF1‐high samples. [file HUMU-2026-9554600-s002.pdf]
