# Supplementary material for: SRSF1/Mcl‐1 Axis Drives Apoptosis Evasion and Shapes the Immune Microenvironment to Promote Gastric Cancer Progression
Source: Hum Mutat. 2026 Jun 10;2026:9554600. doi: 10.1155/humu/9554600 (PMC13254220; doi:10.1155/humu/9554600)
Supplement: Supplementary file 2 — Supporting Information 2. Table S1: shRNA sequences targeting different regions of SRSF1 coding sequence (CDS). Table S2: qRT‐PCR primer pairs targeting multiple genes (forward and reverse sequences). [file HUMU-2026-9554600-s001.docx]

1. **Supplementary Table S1**: shRNA Sequences Targeting Different Regions of SRSF1 Coding Sequence (CDS)

|  | shRNA sequence |  |
| --- | --- | --- |
| shRNA1 | 5’-GAAGCAGGTGATGTATGTTAT-3’ | Y29189 |
| shRNA2 | 5′-ACTTACCTCCAGACATCCGAA-3′ | Y29190 |
| shRNA3 | 5’-TGGTCGCGACGGCTATGATTA-3′ | Y29191 |

| Gene(H) | Prime | Product length |
| --- | --- | --- |
| SRSF1 | F: CATAACTCATCATTCCCCAGA | 101 |
|  | R: CCTCTCCAATCCAAACCTT |  |
| Caspase9 | F: GGAAGAGCTGCAGGTGGAC | 75 |
|  | R: TCCTCGATCATATGGGGCCT |  |
| BAK1 | F: TCCAGACCATGTTGCAGCAC | 101 |
|  | R: GCCCCAATTGATGCCACTCT |  |
| Caspase3 | F:ATGGGTGCTATTGTGAGGCG | 117 |
|  | R: TCACGGCCTGGGATTTCAAG |  |
| MCL1 | F: GCGACTTTTGGCCACCG | 244 |
|  | R:GAGAGTCACAATCCTGCCCC |  |
| GAPDH | F:GGAAGGAAATGAATGGGCAGC | 148 |
|  | R:TAGGAAAAGCATCACCCGGAG |  |

1. **Supplementary Table S2:** qRT-PCR Primer Pairs Targeting Multiple Genes (Forward and Reverse Sequences)
